# Who Decides What Is Prebiotically Plausible? The Risks of Premature Constraints in Origin-of-Life Research

**DOI:** 10.3390/life15111650

**Published:** 2025-10-22

**Authors:** Simon H. J. Eiby, Tue Hassenkam

**Affiliations:** Globe Institute, University of Copenhagen, DK-1350 Copenhagen, Denmark; tue.hassenkam@sund.ku.dk

**Keywords:** origin of life, prebiotic chemistry, early Earth, prebiotic plausibility, RNA world, messy environments, research diversity

## Abstract

The origin of life is the ultimate scientific puzzle. The leap in complexity from inanimate matter to even the simplest known organisms is overwhelming, and the transition from simple chemistry to life is best viewed as a long, directionless pathway. So, how did life arise de novo from simple chemical molecules? The chemical space of potential reactants, catalysts and inhibiting agents is vast, while our knowledge of prebiotic conditions is limited. This makes it difficult to assess whether reaction pathways are prebiotically plausible. Origin-of-life research is therefore inherently speculative and shaped by competing schools of thought. Prebiotic plausibility should inform discussion and exploration, but not impose undue constraints based on personal preferences. Genuine progress is achieved through openness to diverse approaches and scenarios, ensuring that a broad spectrum of studies and their underlying rationales, assumptions, and methodologies are visible and explored.

## 1. Introduction

The earliest traces of life are 3.7 billion years old [1] while liquid water was present on Earth as early as 4.4 billion years ago [2], implying that life emerged within this interval. However, geological and biological records offer only limited insight into exactly when and where life emerged, as well as how it occurred. Geological processes have erased physical traces of prebiotic stages, and phylogenetic records trace back only to the last universal common ancestor (LUCA), which is already a relatively complex organism with molecular machinery resembling that of modern life [3]. This has led us to prebiotic chemistry, which aims to understand and recreate how living systems could emerge from simple abiotic molecules under presumed prebiotic conditions. Competing hypotheses on the chemical emergence of life focus on which defining feature emerged first, such as replication, metabolism, or compartmentalization. Replication-first models propose that self-replicating molecules initiated primitive evolution, progressively increasing chemical complexity and functionality [4], while dismissing spontaneous prebiotic metabolic networks as implausible [5]. Metabolism-first models contend that the intrinsic biochemistry of life serves as a guide to its origins, arguing that primitive prebiotic metabolic networks were supplied by geochemical processes and could not have arisen through blind evolution alone [6]. Compartment-first models stress the necessity of physical boundaries and spatial organization of molecules but are often integrated into replication [7] or metabolism-driven [8] frameworks as essential supporting elements. While differing in focus, all models are required to tackle common chemical challenges, including how informational and functional polymers could have emerged under prebiotic conditions.

## 2. The Uncertainty of Prebiotic Conditions

“Prebiotic conditions” is an evolving concept shaped by ongoing discoveries. Conditions on early Earth changed dramatically between its formation and the earliest evidence of life [9], and we do not know the exact timing of life’s emergence within this interval. Moreover, our understanding of the conditions on early Earth is based on limited data points. Early Earth likely hosted a wide range of local environments, including submarine hydrothermal vents, subaerial geothermal pools, icy environments, and hybrid environments in between, each offering different kinds of dynamic conditions. Temperatures could range from freezing to over 100 °C, pH from acidic to alkaline, and redox conditions from highly reducing to more oxidizing. Submarine alkaline hydrothermal vents, powered by geochemical serpentinization, provide unique redox chemistry that can drive carbon fixation and potentially primitive metabolic networks [8]. In contrast, subaerial geothermal pools are exposed to energetic radiation and undergo repeated wet/dry cycles that concentrate solutes and can promote prebiotic synthesis and non-enzymatic polymerization [10]. Finally, icy environments enhance chemical stability and trap solutes in concentrated brines within the ice, forming primitive compartments and can support RNA replication [11]. The wide range of possible prebiotic conditions and local environments makes it arbitrary to pinpoint the conditions fostering life’s origin and should be kept in mind when evaluating new findings in prebiotic chemistry. Rather than judging based on personal preferences about “prebiotic conditions”, emphasis should be placed on ensuring the transparent reporting of findings, methods, and rationale to enable valid comparison, replication, and further investigation.

Another key question related to prebiotic conditions concerns the availability of organic materials on early Earth. Analyses of meteorites [12] and asteroids [13] have revealed various organic molecules, including potential building blocks of life, indicating that such compounds were present on the prebiotic Earth. However, their abundances relative to other materials and local environmental contexts remain uncertain. Laboratory simulations of prebiotic chemistry provide a controlled way to explore reaction pathways and assess how reactants and products could contribute to the origin of life. This includes the formation of relevant building blocks of life and their assembly into informational and functional polymers.

## 3. Prebiotic Chemistry in the Laboratory

A chemistry laboratory bears little resemblance to the early Earth, both in terms of the chemical complexity of reactions and the timescales over which reactions occur. Nevertheless, carefully controlled experiments can be useful to explore chemical possibilities. While such experiments show that a product can be formed under controlled, and presumed prebiotic, conditions, they do not guarantee that nature would have followed the same path, nor that this product would have been abundant in the prebiotic mix. Laboratory simulations of prebiotic chemistry are inherently biased, since their boundaries and parameters are set by the experimenter. They are often designed to favor a desired outcome, for example, by using pure, highly concentrated, chemically activated reactants and carefully controlled step changes in conditions such as pH and temperature. Under such controlled setups, it appears possible to produce virtually any compound, which can give a misleading impression of prebiotic plausibility. As previously emphasized, clearly specifying the prebiotic scenario being simulated, along with all experimental interventions, is essential for evaluating plausibility and ensuring transparency [14].

Abiotic pathways to RNA have drawn much attention due to RNA’s dual role in life as both a genetic carrier and a catalyst (ribozymes), giving it the potential to ‘work alone’ in early life forms [4]. We do not know if RNA is an abiotic or biotic invention, and whether its formation depended on primitive metabolic networks or not. If prebiotic metabolic networks were present, they may have supplied nucleotides, the building blocks of RNA, but no experimental evidence currently supports this. Synthesizing nucleotides de novo from simple prebiotic molecules is considered challenging [15]. However, experimentally demonstrated reaction networks yielding canonical nucleotides suggest that prebiotic reservoirs of such building blocks are not impossible [16,17]. The complexity of RNA has led researchers to propose that simpler polymers may have preceded RNA and gradually transitioned into it over time [18,19]. However, no obvious precursor candidate that is significantly simpler than RNA has yet been identified. The emergence of RNA from a heterogeneous mixture of nucleotides has been suggested to occur through chemical selection via cycles of replication [20]. Whether RNA originated directly from canonical nucleotides, gradually emerged from heterogeneous nucleotide mixtures, arose from entirely different polymers, or followed another pathway remains an open question. All these approaches are equally relevant to explore further and can inform open and relevant discussions of RNA-first vs. RNA-later scenarios. In our opinion, decisive statements about the emergence of RNA seem premature and should not be used to dismiss new findings and constrain exploration. Furthermore, the emergence of informational and functional polymers such as RNA should be regarded as a unifying challenge relevant to all origin-of-life scenarios instead of one mostly confined to replication-first.

The non-enzymatic polymerization of nucleotides into long RNA strands is widely regarded as a central challenge in prebiotic chemistry. Polymerization is energetically uphill, and RNA is inherently unstable in aqueous solution where it readily hydrolyzes back into nucleotide monomers. Despite these difficulties, several approaches have been reported to achieve the polymerization of nucleotides non-enzymatically. These include chemically activated nucleotides (triphosphates) incubated with rock glasses in aqueous solutions at neutral pH and room temperature [21], hot wet/dry cycling of non-activated nucleotides at acidic conditions [22], and the drying of intrinsically activated 2′–3′-cyclic nucleotides at mildly warm to hot alkaline conditions [23]. Typically, these approaches rely on high concentrations and purity of reactants under specific, controlled conditions, producing mixed 2′–5′ and 3′–5′ phosphodiester linkages and relatively low yields, particularly for non-activated nucleotides. Efficient non-enzymatic RNA copying through templated primer extension using phosphorimidazolides (activated nucleotides) has been reported [24]. While these approaches to obtain RNA polymerization and copying may not reflect the processes involved in the emergence of life, they are highly relevant, as they establish baselines for the chemical possibilities using conceptually simple and direct precursors. We cannot say which type of nucleotides or activation is most relevant to study and further research on non-enzymatic RNA polymerization and copying is needed, both through new and creative approaches and by extending existing ones. A key milestone is to show that a large RNA pool can be non-enzymatically synthesized and sustained, from which advantageous functions can emerge and evolve through selection. This would directly connect prebiotic RNA synthesis with in vitro evolution studies.

## 4. Prebiotic Plausibility

Prebiotic plausibility is a key consideration in origin-of-life research, but is difficult to define and measure. One of the pioneers of prebiotic chemistry, Leslie Orgel, proposed three general constraints for prebiotic synthesis pathways: (1) the starting materials could have been present in adequate amounts on the prebiotic Earth, (2) reactions occur in water or without a solvent, and (3) the reactions produced significant yields [4]. However, Orgel also noted that ‘prebiotic’ is an ‘elastic term’, and that deciding plausibility is partly up to the proposer, making enforcement of these constraints difficult. While these constraints provide a useful foundation for discussing new findings, they must be applied with caution. In ‘clean chemistry’ laboratory experiments, the starting materials and their concentrations may be argued to be prebiotically plausible, but this does not automatically mean that the reactions, intermediates, or products are themselves prebiotically plausible. Furthermore, product yields reflect the timescales of laboratory experiments, typically hours to days, which may be far shorter than those relevant under prebiotic conditions. Over much longer prebiotic timescales, yields could differ substantially, potentially favoring reactions that are negligible or undetectable in short-term laboratory studies. Slow but steady accumulation of reactants, intermediates, and products over geological timescales can shift dominant pathways and increase long-term yields. Judging plausibility in a field with so many uncertainties is inevitably subjective and prone to bias. Although many studies emphasize prebiotic plausibility as a key factor guiding research and discussion [15], it is difficult to imagine a shared consensus on what counts as plausible. Overemphasizing plausibility can polarize debates into “right versus wrong” arguments, rather than highlighting how different findings complement one another.

Deamer has argued that we should not restrict our studies to ‘clean chemistry’ in the laboratory, as life did not emerge under such controlled conditions but in messy natural environments [25]. In such natural environments, numerous environmental factors, such as co-existing solutes and varying physical and chemical conditions, would have influenced reactions pathways, producing complex reaction mixtures. However, order can still emerge in such contexts. Deamer emphasizes that processes like self-assembly and selective adsorption can concentrate and preserve certain molecules within the disorder. Conducting prebiotic chemistry experiments in natural environments is inherently challenging due to the ubiquitous presence of life. Nevertheless, it is possible to introduce complexity and a degree of messiness in laboratory simulations, for example, by using multiple simple reactants and allowing reactions to proceed over extended timescales under naturally fluctuating conditions, without abrupt step changes from interventions or the purification of intermediates. Messiness should not be treated as a complication to remove, but as a key factor guiding prebiotic molecular selection. Approaches to study messy chemical systems have been proposed, including routinely measuring bulk properties and dynamical behavior [26] and conducting automated long-term experiments [27]. When organic molecules are mixed in the laboratory and allowed to react uncontrolled under heat or other conditions, presumed prebiotic, the outcome is rarely a single desired product. Instead, these reactions typically yield a messy, complex mixture of molecules referred to as prebiotic clutter [18]. Chemistry, left uncontrolled, tends toward devolution rather than evolution. The question then arises: how did the building blocks of life escape this fate and start forming ordered structures? Natural catalytic networks, compartments, organizing agents, and self-assembly may have helped pre-select and protect specific molecules, allowing them to persist and participate in further chemistry. Thus, metabolism-first and compartment-first may better accommodate life’s emergence in messy environments, while replication-first face greater challenges in explaining the initial selection and protection of specific molecules. However, competing frameworks should not be seen as opposing but as offering complementary perspectives that, together, can guide the development of coherent and plausible solutions. In our opinion, escaping the prebiotic clutter is a key step from chemistry to biology. In addition to pursuing high yields of single products in controlled laboratory experiments, we should also focus on pathways and products that can emerge from the prebiotic clutter in deliberate messy environments. After all, the only known origin of life occurred not in a laboratory but from the prebiotic clutter.

## 5. Outlook

Prebiotic plausibility should not be treated as absolute, and we should remain open to diverse perspectives and alternative approaches while building on our understanding. The quality of research should primarily be judged by the clarity and robustness of the methods, results, and reasoning. Disagreement should encourage openness and genuine engagement, creating a foundation for inclusive, thoughtful discussions. The stronger the disagreement, the greater the need for careful attention to understanding opposing views. The availability and visibility of studies exploring a wide range of hypotheses and approaches are essential for open and unbiased discussion. From this, an inclusive and diverse community can thrive, fostering an exciting future for origin-of-life research.

## Data Availability

No new data were created or analyzed in this study. Data sharing is not applicable to this article.
